# Gold Nanoparticles as Targeted Drug Delivery Systems for Liver Cancer: A Systematic Review of Tumor Targeting Efficiency and Toxicity Profiles

**DOI:** 10.3390/ijms26167917

**Published:** 2025-08-16

**Authors:** Meda Cosma, Teodora Mocan, Cristian Delcea, Teodora Pop, Ofelia Mosteanu, Lucian Mocan

**Affiliations:** 1 Department of Physiology, “Iuliu Hatieganu” University of Medicine and Pharmacy, 400012 Cluj Napoca, Romania or manolea.meda@umfcluj.ro (M.C.); teodora.mocan@umfcluj.ro (T.M.); 2 Department of Forensic Medicine, “Iuliu Hatieganu” University of Medicine and Pharmacy, 400012 Cluj Napoca, Romania; 3 3rd Surgery Clinic, “Iuliu Hatieganu” University of Medicine and Pharmacy, 400012 Cluj Napoca, Romania; teodorapop@gmail.com (T.P.); ofeliamosteanu@gmail.com (O.M.); lucian.mocan@umfcluj.ro (L.M.)

**Keywords:** liver cancer, gold nanoparticles, drug delivery, functionalization

## Abstract

Hepatocellular carcinoma (HC) ranks as the fifth most prevalent form of cancer among humans and is a significant contributor to cancer-related deaths. During the latest year, an interesting scientific fascination arose around gold nanoparticles (AUNPs) following the recovery of their remarkable properties. Some studies suggest that AUNPs can enhance drug targeting in cancer treatment and reduce its toxicity. The major purpose of this paper is to systematically review the effectiveness, safety, and prospective mechanism of gold nanoparticles in delivering drugs for liver cancer treatment. Comprehensive research was conducted using major scientific databases (i.e., PubMed, Web of Science, and Scopus) to identify studies focusing on AUNPs in drug delivery systems. We mainly focused on studies that specifically analyzed liver cancer. The current results of the systematic review show that the application of gold nanoparticles (AUNPs) in liver cancer drug delivery enhances drug targeting to liver tumors. This efficient factor improves the bioavailability and elevates the therapeutic index of chemotherapeutic agents in treatment. This systematic review highlights the significant potential of AUNPs to increase the delivery of drugs for liver cancer treatment effectively. The major findings indicate that AUNPs improve the targeting and bioavailability of chemotherapeutic agents, enhancing therapeutic outcomes such as tumor suppression and improved survival rates. While the results of this review are encouraging; however, further research is necessary to ensure the safety and efficacy of AUNPs in clinical settings. Human trials must address concerns regarding long-term toxicity and regulatory approval.

## 1. Introduction

Hepatocellular carcinoma (HC) is identified as the fifth most prevalent type of cancer among the human population. Annually, approximately 800,000 new cases of liver cancer are diagnosed [1]. Some risk factors for HC are well-known, and the most common are hepatitis B or C, excessive alcohol consumption, and a condition known as non-alcoholic fatty liver disease [2]. The treatment of liver cancer is complex and presents significant challenges. The surgical resection of malignant tumors, which can be applied in 10–30% of cancer patients, is the only curative option for treatment [3]. Individuals diagnosed with HC frequently encounter adverse outcomes following chemotherapy and radiotherapy, underscoring the urgent need for innovative and more efficacious therapeutic approaches [4,5].

**Figure 1:**
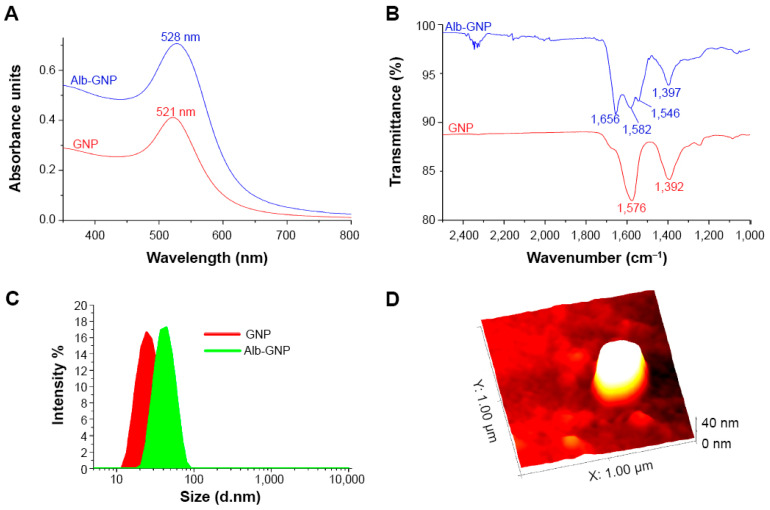
Characterization of Albumin-functionalized Gold Nanoparticles with applications in photothermal therapy of liver cancer. Notes: (**A**) UV-Vis spectra of AUNPs (red line) and BSA-AUNP samples (blue line). (**B**) FT-IR spectra for AUNP (red) and BSA-AUNP (blue) in the 2500–1000 cm^−1^ region. (**C**) DLS size distribution curves for AUNP (red) and BSA-AUNP (green). (**D**) AFM image of a single BSA-AUNP nanoparticle. Abbreviations: AUNP, gold nanoparticle; BSA, bovine serum albumin; IR, infrared; UV-Vis, ultraviolet visible; DLS, dynamic light scattering; AFM, atomic force microscopy. Republished with permission from reference [8].

The science of surface functionalization chemistry has led to the development of a diverse spectrum of versatile bio-nanomaterials with potential applications in oncology [6,7]. Plasmonic (noble metal) nanoparticles exhibit tunable electronic and specific optical properties, enhancing their importance in therapeutic applications, such as laser nanomediated photothermolysis for cancer treatment (Figure 1) [8].

Nanoparticles represent a promising approach for cancer treatment [9]. They have great potential to improve drug bioavailability and cross various biological barriers [10]. Their impressive compatibility with biological systems, ease of surface modification, and capacity to enhance drug delivery are of high importance in pharmacology [11].

Gold nanoparticles (AUNPs) are extensively studied as anticancer therapeutic agents [12] due to their tunable sizes and morphology, biocompatibility, and minimal toxicity [13,14].

AUNPs may be synthesized utilizing various physical or chemical techniques. Their size typically varies from 1 to 100 nm [15]. The unique physical and chemical properties that arise from the nano-ionization of these structures make them suitable for use in oncology. A particularly characteristic of metal nanoparticles is the phenomenon of surface plasmon resonance (SPR) following photon excitation [8]. The SPR effect enables AUNPs to generate a spectrum of colors, enhancing their application in colorimetric biosensors employed in diagnostic medicine [16].

Gold nanoparticles (AUNPs) can be engineered to selectively target cancer cells, thereby allowing for the controlled release of chemotherapeutic agents [17]. Consequently, AUNPs exhibit considerable potential in developing novel therapeutic strategies for hepatic carcinoma.

Conventional therapeutic interventions, such as chemotherapy and surgical resection, remain predominant treatment methods for liver cancer; however, these approaches are not without side effects. They lack specificity and pose systemic toxicity risks [18]. Chemotherapeutic agents are associated with significant adverse effects due to their non-selective mechanisms of action [19]. Moreover, the treatment of liver cancer presents considerable medical challenges, such as drug resistance, thereby diminishing the efficacy of standard therapeutic regimens [5].

Recently, AUNPs have been of great interest in HC treatment due to their potential to enhance drug delivery systems [20]. Preliminary studies suggest that AUNPs may improve drug targeting to cancerous tissues while reducing systemic toxicity. However, further comprehensive investigations are needed to evaluate their long-term safety, overall effectiveness, and the mechanisms by which they optimize drug delivery [21].

This manuscript mainly aimed to conduct a systematic review of the efficacy, safety, and prospective mechanisms associated with gold nanoparticles in the context of drug delivery for liver cancer therapy. Through a rigorous analysis of the existing literature, this paper elucidates insights into AUNP-based HC therapeutic systems and identifies research directions that need further investigation.

## 2. Methods

### 2.1. Search Strategy and Information Sources

Extensive empirical investigations were performed using leading scientific repositories (i.e., PubMed, Web of Science, and Scopus) to identify scholarly works focusing on AUNPs within drug delivery systems. Our primary focus was on studies that specifically examined liver cancer. The search aimed to acquire peer-reviewed articles published between 2020 and 2024. The keywords employed in the search included “gold nanoparticles,” “drug delivery systems,” “liver cancer,” “chemotherapy,” “targeted therapy,” and “bioavailability enhancement.”

Boolean operators were employed to amalgamate terms and optimize the search outcomes. This methodological step was undertaken to ensure that the identified studies were distinctly pertinent to AUNPs and their application in treating liver cancer.

### 2.2. Inclusion and Eligibility Criteria

The papers related to the application of gold nanoparticles as a drug delivery platform for treating liver cancer were analyzed. Only peer-reviewed studies employing in vitro, in vivo, and clinical trial methodologies were included. In accordance with the inclusion criteria, the selected studies showed quantifiable treatment outcomes, such as tumor regression, survival rates, bioavailability enhancements, or drug delivery efficiencies. Furthermore, only studies published between 2020 and 2024 in the English language were included.

### 2.3. Exclusion Criteria

Research papers focusing on other types of nanoparticles (such as silver and iron oxide nanoparticles) or on malignancies unrelated to hepatic conditions were removed from our analysis. Moreover, review articles, editorials, and letters were not included. Studies lacking therapeutic assessment within cancer model systems were also excluded.

### 2.4. Data Extraction and Risk of Bias

**Figure 2:**
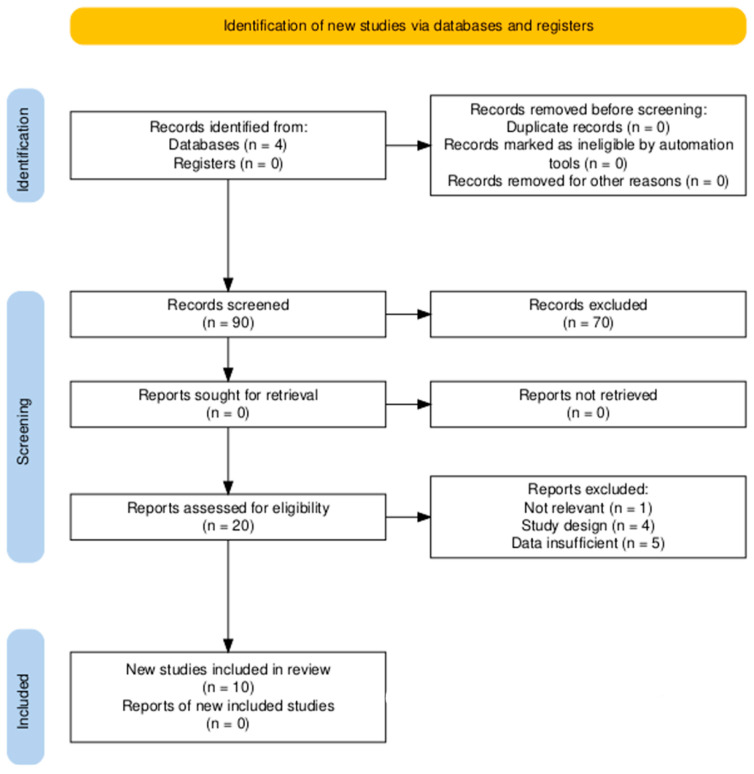
Prisma flow chart.

The process of data extraction and analysis was performed according to the PRISMA guidelines (Figure 2) to fully ensure reproducibility of the findings, following defined inclusion and exclusion parameters. Parameters such as study design (in vitro, in vivo, and clinical), characteristics of AUNPs (dimensions and targeting mechanisms) (Table 1), and outcomes, such as bioavailability, reductions in tumor size, rates of survival, and toxicity profiles (Table 2), were analyzed and included in this paper.

### 2.5. Synthesis of the Results

The extracted data were synthesized using a qualitative approach due to the variability in study designs, nanoparticle sizes, drugs delivered, and outcome measures. Although the data were not suitable for a meta-analysis, thematic analysis was used to identify recurring trends in AUNP effectiveness, safety, and targeting mechanisms across the studies. Key findings related to tumor reduction, bioavailability, and toxicity profiles were compared across studies to identify consistencies and discrepancies. These results were categorized into thematic subgroups for discussion, focusing on AUNP-mediated drug delivery efficiency, enhancement of therapeutic indices, and potential toxicity concerns.

## 3. Results

**Figure 3:**
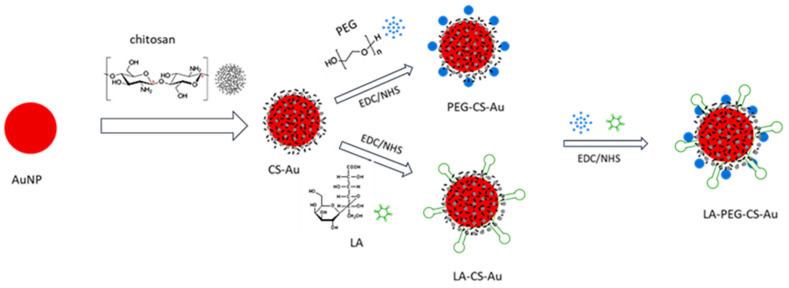
Receptor Targeting Using Copolymer-Modified Gold Nanoparticles for pCMV-Luc Gene Delivery to Liver Cancer Cells In Vitro. AuNPs were synthesized and coated with polymers, including chitosan (CS) and polyethylene glycol (PEG). The targeting moiety, lactobionic acid (LA), was added for hepatocyte-specific delivery. Schematic illustration republished with permission from reference [22].

The current results of the systematic review demonstrate that the application of gold nanoparticles (AUNPs) in liver cancer drug delivery enhances drug targeting to liver tumors. This efficient factor improves the bioavailability and elevates the therapeutic index of chemotherapeutic agents in the treatment. Studies by Zenze and Singh [22] revealed a fivefold increase in gene expression in HepG2 cells due to receptor-mediated endocytosis, which demonstrates the drug delivery efficiency of AUNPs (Figure 3).

**Figure 4:**
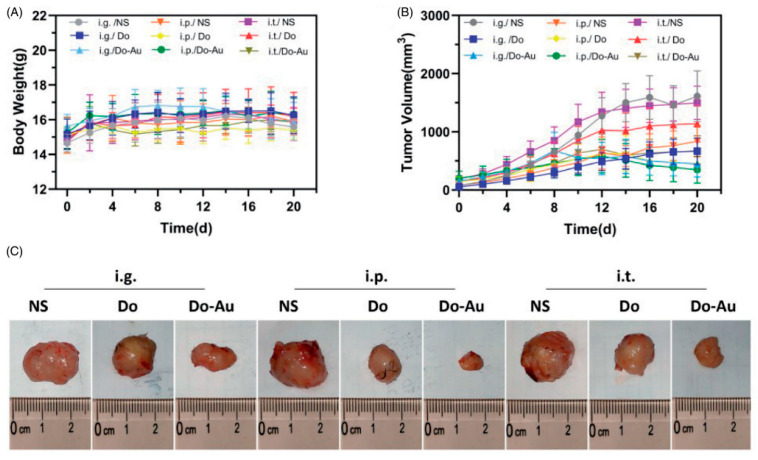
Gold nanoparticle (Do-AuNP) was successfully synthesized from Dendrobium officinale (DO). Do-AuNP had better anti-tumor efficiency compared to DO extraction alone without increasing toxicity in vivo and in vitro. Gross tumor index. (**A**) Body weights during 20 days. (**B**) Time-dependent tumor volume curves of different groups. (**C**) Photographs of tumors from nine different groups. Republished with permission from reference [25].

The investigation conducted by Zhao et al. [25] also revealed substantial reductions in tumor size across both in vitro and in vivo experimental models, thereby underscoring the efficacy of gold nanoparticles (AuNPs) in the targeted treatment of hepatic carcinoma (Figure 4). Additionally, a separate scholarly article by Ding et al. [26] demonstrates that 85.6% of tumor proliferation is impeded via passive targeting mechanisms attributed to enhanced permeability and retention (EPR) effect. This finding underscores the capability of AuNPs to facilitate the accumulation of therapeutic agents at tumor loci, thereby improving overall therapeutic efficacy.

One of AUNPs’ major significances is the increased bioavailability of administered drugs. For example, Nasef et al. [23] observed better pH-responsive release of fluorouracil, resulting in more controlled and sustained drug delivery. This enhances bioavailability, resulting in increased outcomes due to more drug retention and better targeting. Additionally, research by Huang et al. [31] indicates that indocyanine green-conjugated AuNPs (ICG-Au25SG18) improve drug retention in the bloodstream, which increases bioavailability through glutathione (GSH) depletion and further increases drug targeting to tumors.

Improved therapeutic indicators were also observed across the selected studies in a systematic review. Hassanen et al. [27] reported that cisplatin-AUNP conjugates reduce nephrotoxicity compared to free cisplatin, enhancing drug delivery to hepatic tumors meanwhile reducing off-target effects. This approach not only improved safety but also reduced liver enzyme levels, indicating better histological outcomes. In a study by Ding et al. [26], a link was found between AUNP-facilitated drug retention and prolonged survival in mice, resulting in more efficient delivery and lowered toxicity of camptothecin (CPT).

### 3.1. Mechanisms of Action

AUNPs employ various mechanisms to enhance the penetration of pharmacological agents into hepatic cancerous cells. According to the findings presented by Zenze and Singh [22] and Nasef et al. [23], receptor-mediated endocytosis is extensively utilized. Specific ligands, including lactobionic acid, are designed to target receptors such as the asialo-glycoprotein receptor, which is notably overexpressed in hepatic cancer cells, thereby facilitating effective drug assimilation. The process of active targeting is augmented by passive targeting through the enhanced permeability and retention (EPR) effect, as demonstrated in the work by Ding et al. [26]. AUNPs demonstrate a propensity to accumulate within neoplastic tissues due to the inherent permeability of the aberrant vasculature characteristic of tumors. Furthermore, AUNPs contribute to the attenuation of multidrug resistance in hepatic cancer by promoting apoptosis via the induction of oxidative stress. Research conducted by Rajeshkumar et al. [30] indicates that fucoidan-mediated AUNPs instigate oxidative stress, ultimately leading to cell death in HepG2 cells, thus presenting an additional avenue for therapeutic intervention.

### 3.2. Safety and Toxicity

AUNP-mediated drug delivery systems typically exhibit a reduced toxicity profile compared to conventional chemotherapy regimens. Zhao et al. [25] noted the absence of significant hepatic or renal toxicity, corroborated by normal hepatic function markers, such as alanine aminotransferase (ALT) and aspartate aminotransferase (AST). In a similar vein, Ding et al. [5] reported no systemic toxicity, as evidenced by the histological integrity of liver and kidney tissues. Cisplatin, a highly cytotoxic antineoplastic agent, is employed in the treatment of various solid tumors, including liver malignancies. In light of rising incidence rates of hepatic tumors and the non-selective nature of cisplatin toward cancer cells, it is imperative to investigate novel therapeutic modalities to combat these conditions. The present investigation aims to ascertain the capability of gold nanoparticles (AUNPs) in enhancing the hepatotherapeutic efficacy of cisplatin against DENA-induced hepatic tumors while evaluating their potential to mitigate the renal toxicity associated with cisplatin administration. A cohort of forty male Wistar rats was allocated into two distinct groups (*n* = 20): Group A, serving as the negative control, and Group B, representing the model of hepatocellular tumor induction. Four months later, each group was further subdivided into four subgroups as follows: Group (1) received normal saline, Group (2) was administered cisplatin, and Group (3) was treated with AUNPs, whereas Group (4) received AUNPs-cisplatin conjugates. The findings revealed a significant elevation in hepatic and renal function tests, alongside increased oxidant levels and a decrease in antioxidant levels in the DENA-treated group. Profound histopathological changes were discerned in the liver and kidney tissue sections, corroborated by the pronounced immunohistochemical expression of placental glutathione S-transferase, Hep Par 1, and proliferating cell nuclear antigen. Notable enhancements across all quantifiable toxicological parameters were observed in the groups treated with either AUNPs or AUNPs-cisplatin conjugates, a trend not mirrored in the cohort treated solely with cisplatin. It can thus be concluded that AUNPs not only facilitate the targeted distribution of cisplatin to tumor sites but also mitigate the renal toxicity induced by cisplatin, which constitutes a primary concern in cancer treatment. Hassanen et al. [27] also assessed that AUNP-conjugated cisplatin lessens nephrotoxicity in comparison to free cisplatin. Nevertheless, certain research studies have indicated potential adverse effects at elevated concentrations. Abdelsattar et al. [24] further reported that high doses of AUNPs could induce cytotoxicity in non-malignant cells, while Nandhini et al. [28] evaluated that excessive generation of reactive oxygen species (ROS) may underlie the observed toxicity.

The majority of studies converge on the consensus that AUNPs enhance drug delivery and bioavailability. Both Ding et al. [26] and Zhao et al. [25] reported analogous tumor inhibition rates alongside improved survival outcomes.

However, inconsistencies arose due to differences in the size of AUNPs, the types of pharmaceuticals utilized, and the routes of administration. The AUNPs employed in the investigation by Zenze and Singh [22] differed significantly from those utilized by Huang et al. [31], who incorporated smaller nanoclusters. This variation profoundly influenced both distribution and targeting efficacy. Additionally, the methods of delivery had a considerable impact on the outcomes of the respective studies. Zhao et al. [25] demonstrated that intratumoral administration proved to be significantly less effective than either intraperitoneal or intragastric methods due to uneven distribution.

Broadly speaking, nanoparticles (NP) are synthesized from either bulk metals that decompose into diminutive nanostructures or from minuscule units that amalgamate to create nano-sized particles. These methodologies are further employed in physical, chemical, and biological techniques. Gold nanoparticles (AuNPs) are typically produced through the chemical approach, which facilitates meticulous control over their dimensions, morphology, and surface attributes. Various stabilizers and functionalizing agents are integrated to augment the compatibility of AuNPs with biological systems [32].

Nevertheless, escalating apprehensions regarding their toxicity continue to represent a substantial concern within the realm of nanomedicine. The detrimental effects of AuNPs may fluctuate based on factors such as size, morphology, surface charge, and functionalization [14].

AuNPs are minuscule entities with diameters ranging from 1 to 100 nanometers. The exploration of AuNPs’ toxicity encompasses a diverse array of methodologies, including both in vitro and in vivo approaches.

Consequently, modifications in the manufacturing processes of AuNPs, which ensure their effective clearance from biological systems, are paramount to their safe application. A critical factor that has demonstrated potential in mitigating the toxicity of AuNPs is surface modification. For instance, PEGylation of nanoparticles has been shown to enhance biocompatibility by diminishing protein corona formation and prolonging circulation time in the bloodstream.

Hence, the advancement of AuNPs with optimized dimensions and morphology tailored for specific applications is essential to minimize adverse effects [33]. Although extensive investigations have been conducted employing in vitro methodologies, providing valuable insights into the cellular impacts of AuNPs, these may not wholly encapsulate the intricacies of in vivo systems. For example, nanoparticles may exhibit divergent behavior in the presence of the immune system, and their biodistribution may differ in living organisms.

One of the principal challenges associated with AuNPs is their accumulation within biological tissues. In contrast to organic compounds that undergo metabolic processes and are subsequently eliminated, AuNPs can persist within tissues for protracted periods, potentially leading to chronic toxicity. The focus for future advancements in the biochemical domain should be directed toward the development of biodegradable nanoparticles or the identification of methodologies to enhance the excretion of AuNPs [34].

Significant advancements have been made in recent years regarding this topic, leading to an improved understanding of how to tailor the properties of gold nanomaterials for specific applications through controlled fabrication of size and shape, molecular targeting, and targeted surface modification. These techniques enable researchers to assess the interactions of AuNPs with cells, tissues, and organs.

Moreover, comprehensive assays such as Annexin-V/PI staining and Caspase activation facilitate the evaluation of the damage inflicted by nanoparticles through programmed cell death (apoptosis) or uncontrolled cell death (necrosis) [33].

Animal studies, particularly those that utilize murine models and zebrafish, are frequently employed to investigate the biodistribution and long-term effects of AuNPs. Key parameters measured through an in vivo approach encompass tissue accumulation, inflammatory responses, organ functionality, and pathways of excretion [35].

The tissues exposed to nanoparticles (NPs) are meticulously extracted, stained, and subsequently scrutinized for indications of necrosis, inflammation, and morphological alterations.

In a similar vein, gold nanoparticles (AuNPs) with spherical configurations demonstrate reduced toxicity compared to rod-shaped counterparts, due to the variance in their surface area and interaction with cellular membranes. Moreover, the cell death instigated by exposure to AuNPs is intrinsically linked to the dimensions of the nanoparticles. Prior investigations have revealed that 1.4 nm AuNPs precipitate cell death through mechanisms of necrosis, whereas their slightly smaller counterparts, measuring 1.2 nm, elicit cell death via apoptotic pathways [36].

#### Interpretation of the Results

The outcomes from this systematic review underscore the significant and promising potential of AuNPs in the therapeutic intervention of liver cancer. Numerous endeavors, including those conducted by Zenze and Singh [22] and Ding et al. [26], have accentuated advancements in targeting tumors and the delivery of pharmacological agents that utilize AuNPs. The conclusions drawn in this manuscript are consistent with the existing research literature, which similarly highlights the capacity of AuNPs to enhance drug bioavailability and inhibit tumor growth. Chen et al. (2018) [37] articulated that this enhancement is facilitated through both passive and active targeting mechanisms. This systematic review particularly emphasizes the critical importance of receptor-mediated endocytosis and the enhanced permeability and retention (EPR) effect, as both mechanisms play pivotal roles in optimizing drug uptake by hepatocellular carcinoma (HCC) cells. Additionally, these findings propose that AuNPs could be integrated into personalized medicine paradigms, where therapeutic strategies are customized to align with the unique characteristics and requirements of each patient’s tumor.

In contrast to methodologies centered on small particles, such as liposomes and polymeric nanoparticles, AuNPs offer considerable advantages. For example, while liposomes targeting liver cancer exhibit stability, their capacity for drug release is notably limited, as noted in the research conducted by Patra et al. [38]. Conversely, AuNPs provide greater control over drug release dynamics, as demonstrated in studies such as those by Nasef et al. [23], due to pH-responsive techniques that ensure sustained drug delivery. Similarly, polymeric nanoparticles encounter biodegradability challenges; however, AuNPs effectively mitigate these issues by fostering stable and predictable interactions within biological systems [39]. Nonetheless, AuNPs are not devoid of challenges, one of which pertains to their potential long-term toxic effects. Although the majority of studies, including those by Zhao et al. [25], have reported no significant liver or kidney toxicity, the long-term side effects of AuNPs remain inadequately characterized.

Currently, the manufacturing expenses and scalability issues pose substantial barriers to the clinical application of AuNP-based cancer therapies. In addition, regulatory challenges are considerable, as these therapeutic interventions are required to undergo comprehensive safety and efficacy testing. A significant portion of studies referenced in this paper are preclinical investigations, which further complicates their translation into human clinical contexts. Investigative works, such as those by Nandhini et al. [28], are predominantly reliant on in vitro models. These models frequently fail to capture the intricate dynamics of human tumors, thereby constraining the applicability of their findings to authentic clinical scenarios.

Limitations of evidence. Future investigations must prioritize clinical trials to thoroughly delineate the potential of AUNPs in the therapeutic landscape of liver cancer. These trials ought to assess their efficacy and safety in human subjects. Amplified sample sizes are imperative to achieve a more comprehensive understanding of the outcomes. Extended follow-up durations are equally crucial to meticulously evaluate the long-term effects and potential toxicities. Future inquiries should also encompass the exploration of synergistic applications of AUNPs in conjunction with other modalities, such as immunotherapy or radiation. This approach could enhance overall therapeutic effectiveness. It is also of paramount importance to address the manufacturing and regulatory impediments. Collaborative efforts among academia, industry, and regulatory entities will serve as a pivotal factor in the successful integration of AUNP-based therapies into clinical practice.

## 4. Conclusions

This paper looks at how gold nanoparticles (AuNPs) are used to deliver medicine to liver cancer cells. It explains how they work, their safety, and why they matter. We also describe how we picked the studies we reviewed. This helps beginners understand how to search for and evaluate scientific papers.

Gold nanoparticles are tiny particles with big promise in cancer treatment. They can carry medicine to tumors without harming healthy cells. Their surface can be changed easily, and they interact with light in special ways. This makes them useful for both treating and seeing tumors. This review focuses on how they target liver cancer and their safety. We also explain how we selected the studies.

Gold nanoparticles reach tumors in two main ways. First, they build up in tumors naturally because tumor blood vessels are leaky. This is called the EPR effect. Second, they can be linked with molecules that guide them to cancer cells. These molecules stick to certain proteins found only on cancer cells. In liver cancer, one common target is the ASGPR protein. Once in the body, gold nanoparticles enter cells. Changing their surface affects how they move and work inside cells.

Other particles, such as polymer or carbon-based ones, are also used in drug delivery. Polymers break down in the body and are usually safe. Carbon particles carry a lot of drugs. However, gold nanoparticles have a special feature and react to light. This lets doctors trigger them from outside the body. That is a big plus.

Gold nanoparticles absorb and reflect light, especially near-infrared (NIR) light. When hit by NIR, they heat up. This heat can kill cancer cells. It also helps release the drug inside the tumor. This method gives doctors better control over treatment. It also lowers harm to healthy tissue.

The safety of gold nanoparticles depends on size, shape, coating, and dose. Very small ones (under 5 nm) leave the body through the kidneys. Bigger ones may stay in the liver or spleen. Scientists are trying new coatings to make them safer and easier to remove. Some studies suggest that gold particles can cause stress in cells or inflammation. That is why thorough testing and careful design are key before using them on humans.

We searched in PubMed, Scopus, and Web of Science. We only picked papers on gold nanoparticles for liver cancer. We looked for studies with good methods and clear results. We skipped the missing information on targeting or toxicity. We checked how well the particles hit the tumor, how they released drugs, and how safe they were.

Gold nanoparticles can help treat liver cancer in smart ways. They find and attack tumors, show up on scans, and respond to light. However, we still need to learn more about long-term safety. New designs may solve these issues. We hope our review helps others start their research with more confidence. This comprehensive systematic review has underscored the substantial potential of AUNPs for the effective enhancement of drug delivery in liver cancer therapy. The principal findings indicate that AUNPs significantly improve the targeting efficiency and bioavailability of chemotherapeutic agents, which consequently leads to superior therapeutic outcomes, including tumor suppression and elevated survival rates. Research indicating the conjugation of cisplatin with AUNPs has demonstrated a reduction in off-target toxicity associated with liver cancer treatment.

AUNPs possess the capacity for functionalization with specific ligands, enabling active targeting capabilities. This characteristic renders AUNPs particularly advantageous for incorporation into personalized medicine paradigms. AUNPs must be integrated into established clinical practices to mitigate the toxicity frequently linked to conventional chemotherapy while simultaneously enhancing drug efficacy.

Although the findings of this review are promising, additional research is warranted to ascertain the safety and efficacy of AUNPs within clinical environments. Human clinical trials must thoroughly investigate concerns regarding long-term toxicity and the necessary regulatory approvals. By addressing these deficiencies in the existing literature, AUNP-based therapies can potentially emerge as a fundamental component in the treatment of liver cancer, thereby improving patient outcomes and possibly establishing a new standard of care.

## Figures and Tables

**Table 1:** Study Characteristics and Delivery Methods.

Study, Year	Type of Study (In Vitro/In Vivo/Clinical)	Gold Nanoparticle Size (nm)	Drug Delivered	Dosage of Drug	Administration Route	Target Mechanism (e.g., Passive Targeting, Active Targeting)	Tumor Type (Hepatocellular Carcinoma, etc.)
Zenze & Singh, 2024 [22]	In vitro	70–250 nm	pCMV-Luc DNA	Not specified	In vitro (cellular uptake via incubation)	Active targeting using lactobionic acid (LA) for receptor-mediated endocytosis	Hepatocellular carcinoma (HepG2 cells)
Nasef, Khozemy, & Mahmoud, 2023 [23]	In vitro	41.7 nm (AuNPs), 66.7 nm (AAuNPs)	Fluorouracil	95 mg/g loading	Oral delivery (targeting the intestines via pH)	pH-responsive (acidic to neutral pH environment)	Hepatocellular carcinoma (HepG2 cells)
Abdelsattar et al., 2023 [24]	In vitro	22–73 nm (AuNPs@ZnO)	Not applicable (no drug, focus on nanoparticles)	Not applicable	Not specified	Active targeting through nanoparticle-mediated cytotoxicity	Breast cancer (MCF-7), Liver cancer (HepG-2)
Zhao et al., 2021 [25]	In vitro & In vivo	30 nm	Dendrobium officinale extract	2.4 mg/mL	Intraperitoneal (i.p.), Intragastric (i.g.), Intratumoral (i.t.)	Active targeting via immune modulation	Hepatocellular carcinoma (Liver cancer)
Ding et al., 2020 [26]	In vitro & In vivo	5 nm (AuNPs), 50 nm (FeMOF)	Camptothecin (CPT)	7.7% loading content	Intravenous (i.v.)	Passive targeting via EPR effect, active targeting through glucose oxidation, and Fenton reaction	Hepatocellular carcinoma (Liver cancer)
Hassanen et al., 2021 [27]	In vivo	10–17.5 nm	Cisplatin	3 mg/kg (Cisplatin)	Intraperitoneal (ip)	Passive targeting through the EPR effect	Hepatocellular carcinoma (HCC)
Nandhini et al., 2021 [28]	In vitro	5–10 nm	No drug, Enterococcus-mediated AuNPs	10 µg/mL (IC50 for AuNPs)	Direct exposure to cells in vitro (cell culture)	Passive targeting via ROS generation	Hepatocellular carcinoma (HepG2 cells)
Lin et al., 2020 [29]	In vivo	10 nm (Au NPs), 20 nm (Janus Au-MnO NPs)	No drug, Janus Au-MnO nanoparticles	1 mg/mL (vesicles)	Intravenous injection in mice	Active targeting, Sono-chemodynamic therapy (SDT/CDT)	Orthotopic liver cancer (Hepatocellular carcinoma)
Rajeshkumar et al., 2021 [30]	In vitro	21–44 nm	No specific drug, Gold nanoparticles synthesized using fucoidan	Various concentrations (1, 10, 25, 50, 100 μg/mL)	Direct application to HepG2 cells	Active targeting via fucoidan-mediated synthesis	HepG2 (Liver cancer)
Huang et al., 2023 [31]	In vivo	~1.1 nm (Au25SG18 nanoclusters)	Indocyanine Green (ICG)-Conjugated Au NPs	~40 μM	Intravenous Injection	Active targeting via GSH responsiveness	4T1 Triple-Negative Breast Cancer

**Table 2:** Synthesis of the Results.

Study, Year	Key Outcome Measures (e.g., Tumor Size Reduction, Survival Rate)	Toxicity Profile (Impact on Liver, Kidney, etc.)	Bioavailability Improvement	Survival Improvement (%)	Mechanism of Action	Limitations	Duration of Study
Zenze & Singh, 2024 [22]	A five-fold increase in luciferase gene expression in HepG2 cells compared to non-targeted NPs	Well-tolerated in all cells with cell viability >70%	Yes, improved cellular uptake and transgene activity	Not applicable (in vitro)	Receptor-mediated endocytosis via the asialoglycoprotein receptor in HepG2 cells	Limited to in vitro studies; does not address in vivo applications or long-term safety	Not specified (cell viability assessed after 48 h)
Nasef, Khozemy, & Mahmoud, 2023 [23]	97% drug release in 300 min at pH 7.4, strong antimicrobial activity	Reduced cytotoxicity of AAuNPs by combining with AuNPs	Yes, enhanced due to pH responsiveness and polymer matrix	Not applicable (in vitro study)	Controlled drug release through pH change	Limited to in vitro studies, no in vivo testing	Not specified
Abdelsattar et al., 2023 [24]	Reduced cell viability in cancer cells (MCF-7 and HepG-2)	Lower cytotoxicity of AuNPs@ZnO and AAuNPs@ZnO compared to ZnO-NPs in normal cells (HSF)	No drug bioavailability assessed, focus on nanoparticles	Not applicable (in vitro study)	Cytotoxicity through ROS generation and membrane damage	High concentration of NPs required, in vitro only	Not specified
Zhao et al., 2021 [25]	Significant tumor size reduction, reduced cell viability in HepG2 cells	No significant liver or kidney toxicity, ALT, AST, BUN, and CRE normal	Improved through the combination with AuNPs	Not specified	Immune modulation, apoptosis, and increased tumor inhibition	Non-uniform particle size, lower efficacy of the i.t. method	3 weeks
Ding et al., 2020 [26]	Significant tumor suppression, 85.6% tumor growth inhibition	No major systemic toxicity (ALT, AST, BUN, CREA normal), no damage to liver and kidney tissues	Improved circulation time and tumor accumulation	Significantly improved, prolonged survival in tumor-bearing mice	Chemodynamic therapy via Fenton reaction, glucose oxidation by AuNPs to generate H_2_O_2_ and OH∙ for ROS production	Requires further testing in human clinical trials, the phosphate-triggered release system complexity	Not specified
Hassanen et al., 2021 [27]	Significant reduction in liver enzymes (ALT, AST), improved histopathology, reduced nephrotoxicity	Reduced kidney and liver toxicity with cisplatin-AUNP conjugates compared to free cisplatin	Increased targeting and reduced off-target toxicity	Not specified	Conjugated AUNPs improved cisplatin’s delivery to hepatic tumors while reducing renal toxicity	Requires further studies on long-term effects	6 months
Nandhini et al., 2021 [28]	Significant ROS generation, apoptosis induction, and reduced PCNA levels	No in vivo toxicity profile was provided, as it was an in vitro study	Not applicable	Not applicable	ROS-mediated apoptosis, cytochrome c release, and PCNA inhibition	Limited to in vitro; no animal or clinical data	24 h (in vitro)
Lin et al., 2020 [29]	Tumor growth inhibition, enhanced ROS production, and better tumor penetration	Minimal toxicity to healthy tissues, stable blood circulation	Enhanced tumor penetration and accumulation	Not specifically reported	Sono-chemodynamic therapy (SDT/CDT) via ROS generation and cavitation	Limited clinical data; primarily an animal model study	21 days (in vivo)
Rajeshkumar et al., 2021 [30]	Dose-dependent cytotoxic activity against HepG2 cells	Not specified	Improved targeting and stability via fucoidan	Not specifically reported	Induces apoptosis in HepG2 cells through oxidative stress and reduced cell viability	No in vivo studies were conducted; limited to in vitro tests	24 h
Huang et al., 2023 [31]	Enhanced tumor targeting through GSH depletion and prolonged blood retention	No pathological damage to liver, slower hepatobiliary clearance	Increased due to reduced hepatic clearance and prolonged blood circulation	Not reported	GSH depletion slowed the clearance of ICG, enhancing tumor targeting	Limited to animal models, unclear long-term effects on other organs	24 h
